# Supplementary material for: Enterococcus and Eggerthella species are enriched in the gut microbiomes of COVID-19 cases in Uganda
Source: Gut Pathog. 2025 Feb 4;17:9. doi: 10.1186/s13099-025-00678-4 (PMC11796075; doi:10.1186/s13099-025-00678-4)
Supplement: Supplementary file 1 — Supplementary Material 1. [file 13099_2025_678_MOESM1_ESM.pdf]

# Supplemental Material

## Suppl. Table 1 See attached

## Suppl. Table 2

**Supplemental Table 2.** Primer and adaptor sequences

PCR1 primers - amplifying V4 region of 16S rRNA

| | |
|---|---|
| V4_515F_Nextera | TCGTCGGCAGCGTCAGATGTGTATAAGAGACAGGTGCCAGCMGCCGCGGTAA |
| V4_806R_Nextera | GTCTCGTGGGCTCGGAGATGTGTATAAGAGACAGGGACTACHVGGGTWTCTAAT |

Illumina Index adaptors

| | |
|---|---|
| Forward index | AATGATACGGCGACCACCGAGATCTACACXXXXXXXXTCGTCGGCAGCGTC |
| Reverse index | CAAGCAGAAGACGGCATACGAGATXXXXXXXXGTCTCGTGGGCTCGG |

## Suppl. Table 3

**Supplemental Table 3.** AMR genes identified in *Enterococcus* species

| Antimicrobial resistance gene | Drug class | Mechanism | Model | Reads Species |
|---|---|---|---|---|
| AAC(6')-Ii | aminoglycoside antibiotic | antibiotic inactivation | protein homolog | *Enterococcus* (chromosome) |
| dfrE | diaminopyrimidine antibiotic | antibiotic target protection | protein homolog | *Enterococcus faecalis* (chromosome) |
| efmA | fluoroquinolone antibiotic; macrolide antibiotic | antibiotic efflux | protein homolog | *Enterococcus faecium* (chromosome) |
| efrA | fluoroquinolone antibiotic; macrolide antibiotic; rifamycin antibiotic | antibiotic efflux | protein homolog | *Enterococcus faecalis* (chromosome) |
| efrB | fluoroquinolone antibiotic; macrolide antibiotic; rifamycin antibiotic | antibiotic efflux | protein homolog | *Enterococcus faecalis* (chromosome) |
| ErmB | lincosamide antibiotic; macrolide antibiotic; streptogramin antibiotic | antibiotic target protection | protein homolog | *Enterococcus faecalis* (chromosome or plasmid) |
| ErmT | lincosamide antibiotic; macrolide antibiotic; streptogramin antibiotic | antibiotic target protection | protein homolog | *Enterococcus faecium* (chromosome or plasmid) |
| lsaA | lincosamide antibiotic; pleuromutilin antibiotic; steptogramin antibiotic | antibiotic target protection | protein homolog | *Enterococcus* (chromosome); *Enterococcus faecalis* (chromosome) |
| msrC | macrolide antibiotic; streptogramin antibiotic | antibiotic target protection | protein homolog | *Enterococcus faecium* (chromosome) |
| tet(L) | tetracycline antibiotic | antibiotic efflux | protein homolog | *Enterococcus* (chromosome or plasmid); *Enterococcus faecium* (chromosome or plasmid) |
| tet(M) | tetracycline antibiotic | antibiotic target protection | protein homolog | *Enterococcus faecium* (chromosome or plasmid) |
| tet(U) | tetracycline antibiotic | antibiotic efflux | protein homolog | *Enterococcus faecium* (chromosome or plasmid) |

## Suppl. Table 4

**Supplemental Table 4.** USA irritable bowel disease (IBD) studies used for meta-analysis of *Enterococcus* abundance.

| Study name | Citation | Studies by Geography | Locartion | SRA |
|---|---|---|---|---|
| Gut microbiome structure and metabolic activity in inflammatory bowel disease | Franzosa, Eric A et al., 2019 | USA_PRISM_1 | USA | PRJNA400072 |
| Short Article Gut Microbiome Function Predicts Response to Anti-integrin Biologic Therapy in Inflammatory Bowel Diseases | Ananthakrishnan, Ashwin N et al., 2017 | USA_PRISM_2 | USA | PRJNA384246 |
| Gastrointestinal Surgery for Inflammatory Bowel Disease Persistently Lowers Microbiome and Metabolome Diversity | Fang, Xin et al., 2021 | USA_QIITA_11546 | USA | PRJEB38352 |
| Multi-omics analyses of the ulcerative colitis gut microbiome link Bacteroides vulgatus proteases with disease severity | Mills, Robert H et al., 2022 | USA_QIITA_11549 | USA | PRJEB42151 |
| Dietary manipulation of the gut microbiome in inflammatory bowel disease patients: Pilot study | Olendzki, Barbara et al., 2022 | USA_umass | USA | PRJNA642308 |
| Overrepresentation of Enterobacteriaceae and Escherichia coli is the major gut microbiome signature in Crohn's disease and ulcerative colitis; a comprehensive metagenomic analysis of IBDMDB datasets | Khorsand, Babak et al., 2022 | USA_hmbp | USA | PRJNA400072 |

**Suppl. Fig 1**

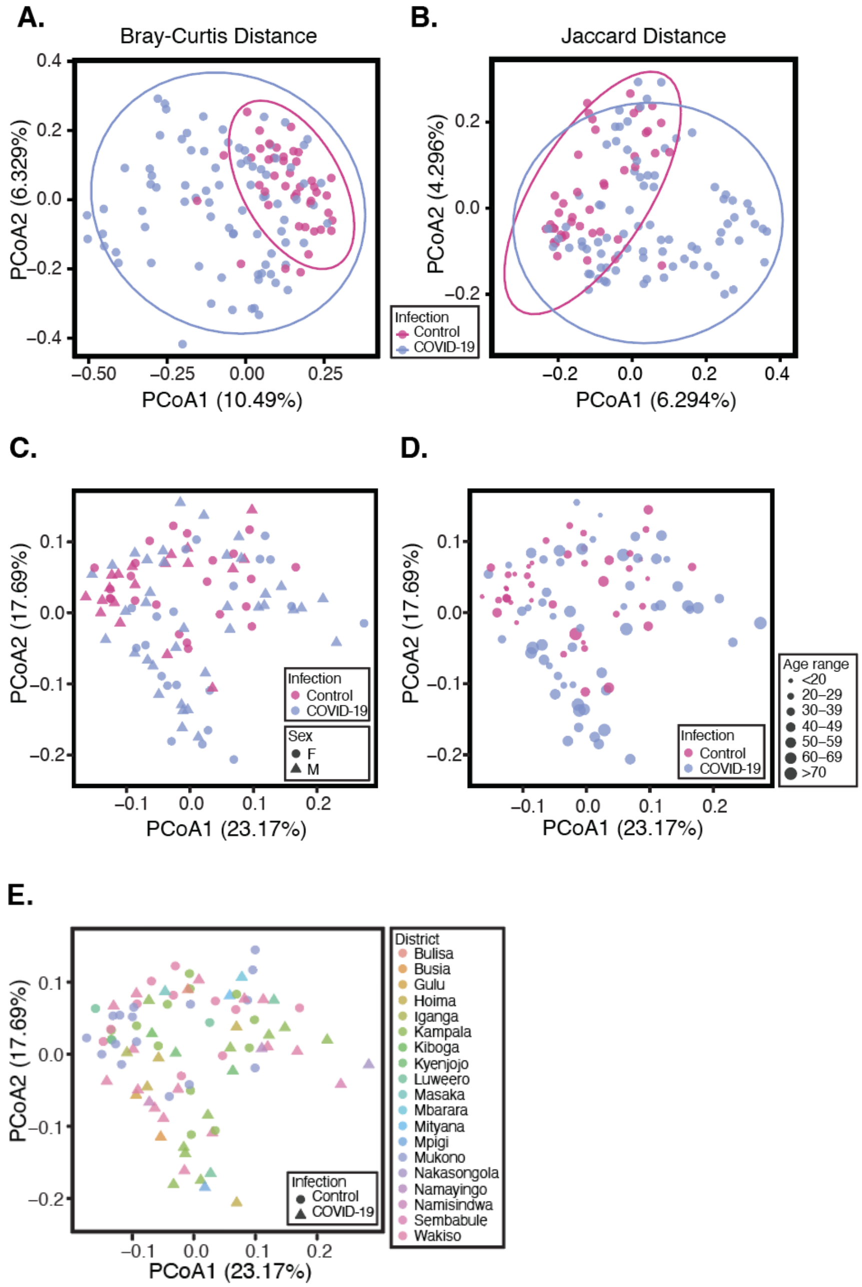


**Supplemental Fig. 1. A.** Principal coordinate analysis (PCoA) calculated using Bray-Curtis distance ($p < 0.01$, PERMANOVA; $p < 0.01$, PERMDISP) and **B** Jaccard distance ($p < 0.01$, PERMANOVA; $p < 0.01$, PERMDISP). PCoA using the weighted UniFrac distance metric is shown **C** for males and females ($p = 0.374$, PERMANOVA; $p = 0.622$, PERMDISP), **D** age range ($p < 0.01$, PERMANOVA; $p < 0.01$, PERMDISP), and **E** district of residence ($p = 0.137$, PERMANOVA; $p = 0.207$, PERMDISP).

**Suppl. Fig 2.**

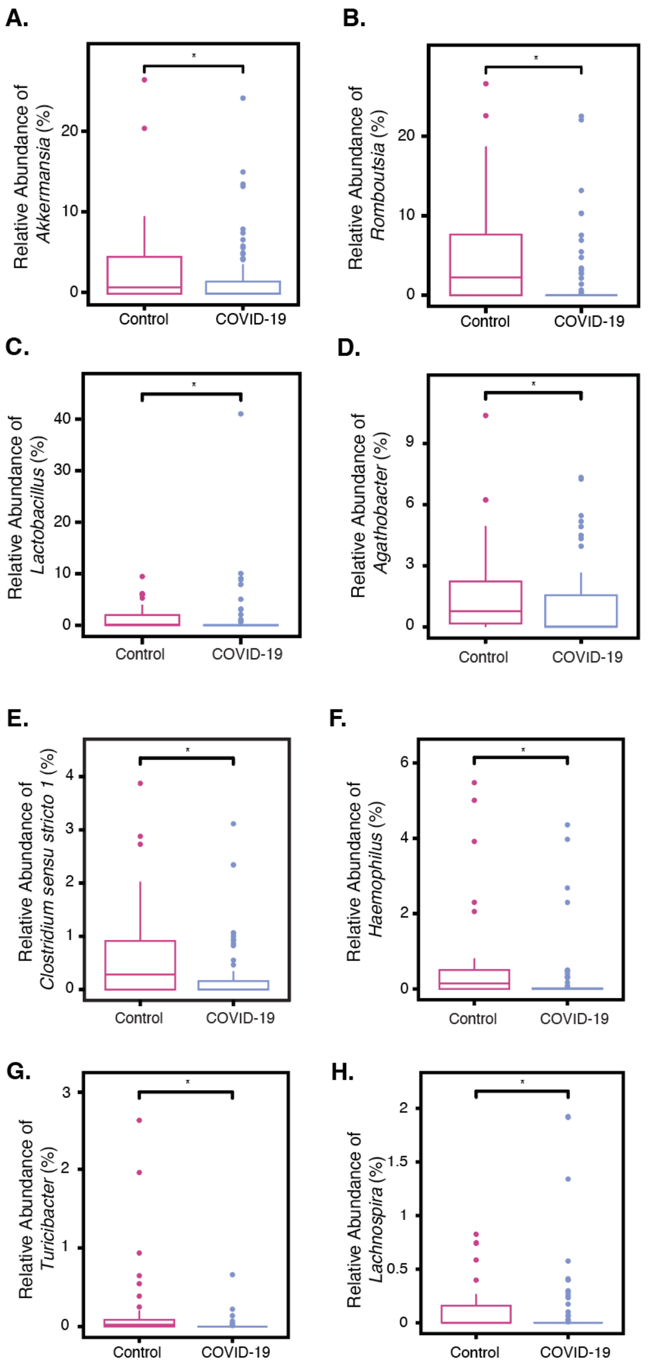


**Supplemental Fig. 2** Genera differentially abundant between COVID-19 cases and controls as determined by ANCOM-BC and MaAsLin2. The relative abundance of **A** *Akkermansia* species, **B** *Romboutsia* species, **C** *Lactobacillus* species, **D** *Agathobacter* species, **E** *Clostridium sensu structo 1* species, **F** *Haemophilus* species,**G** *Turicibacter* species, and **H** *Lachnospira* species (**A-F** padj* < 0.05, Holm test, **G-H** padj* < 0.05 Linear mixed model) in positive COVID-19 individuals and asymptomatic individuals.

**Suppl. Fig. 3**

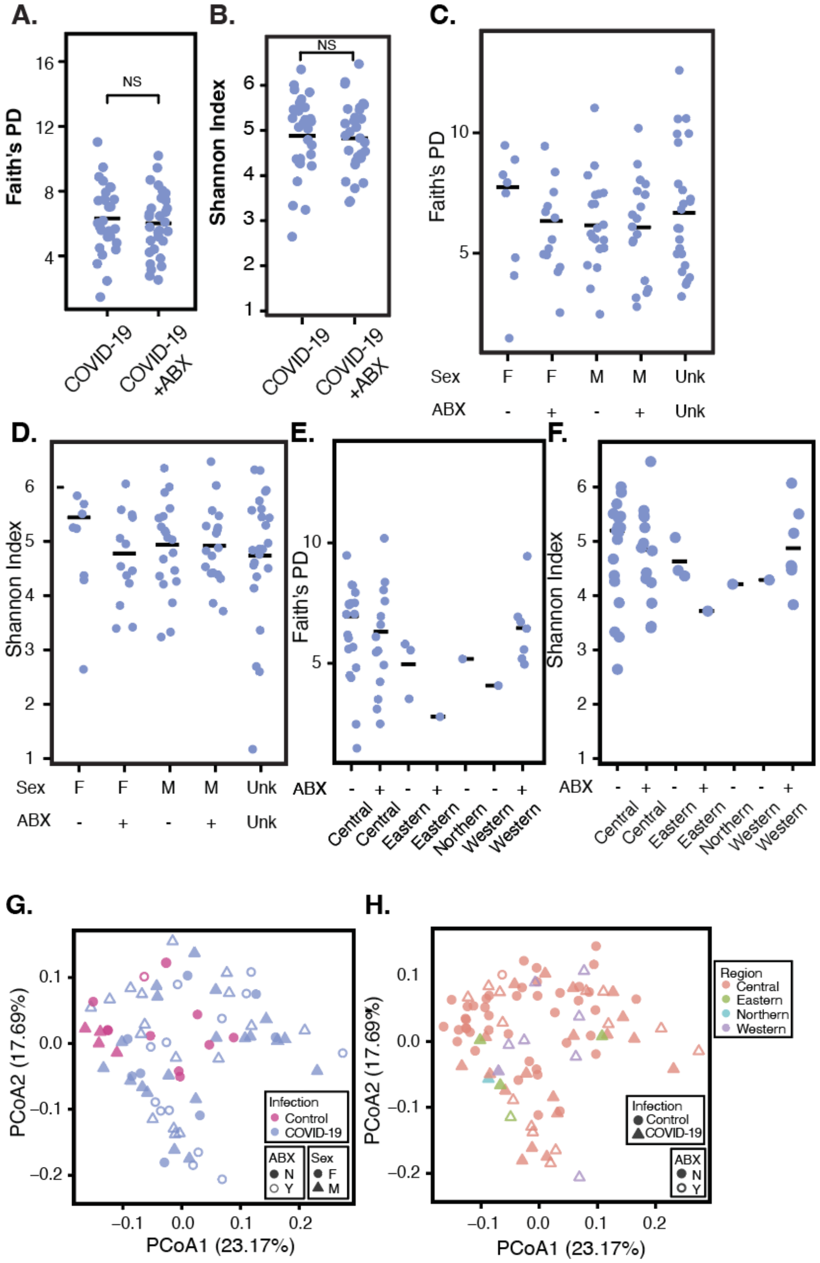
A.
B.
C.
D.
E.
F.
G.
H.
Faith's PD
Shannon Index
NS
COVID-19
COVID-19 +ABX
Sex
ABX
F
M
Unk
Central
Eastern
Northern
Western
PCoA1 (23.17%)
PCoA2 (17.69%)
Infection
Control
COVID-19
ABX
N
Y
Sex
F
M
Region
Central
Eastern
Northern
Western

**Supplemental Fig. 3** The gut microbiome of individuals with COVID-19 with antibiotic exposure is not significantly different than individuals with COVID-19 without antibiotic exposure. Alpha diversity is shown using **A** Faith's Phylogenetic Diversity (PD) and **B** the Shannon Index (p = 0.541 Faith's PD, p = 0.6897 Shannon) (ABX = antibiotics, not significant - NS, Wilcoxon). Alpha diversity based on sex and antibiotic exposure **CD** (p = NS Faith's PD, p = NS Shannon, Hochberg) and geographical region and antibiotic exposure **EF** (p = NS Faith's PD, p = NS Shannon, Hochberg). Principal cooordinate analysis (PCoA) calculated using theWeighted UniFrac distance metric is shown for sex and antibiotic exposure of COVID-19 positive individuals **G** (p = NS, PERMANOVA; p = NS, PERMDISP) and geographical region and antibiotic exposure of COVID-19 positive individuals **H** (p = 0.383, PERMANOVA; p = 0.441, PERMDISP) (ABX = antibiotics, Unk = Unknown, not significant - NS).

**Suppl. Fig. 4**

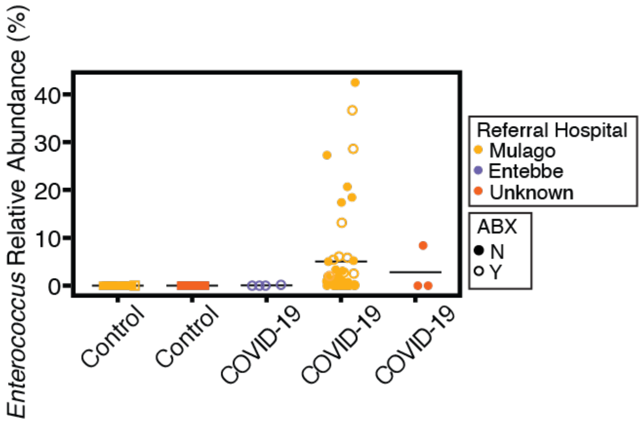


**Supplemental Fig. 4** *Enterococcus* abundance is not dependent on antibiotic exposure at a given hospital (NS, Wilcoxon test). Although *Enterococcus* positive patients were primarily found at Mulago Hospital, the small number of patients at Entebbe did not allow for a comparison in abundance between locations. (NS = not significant).

**Suppl. Fig. 5**

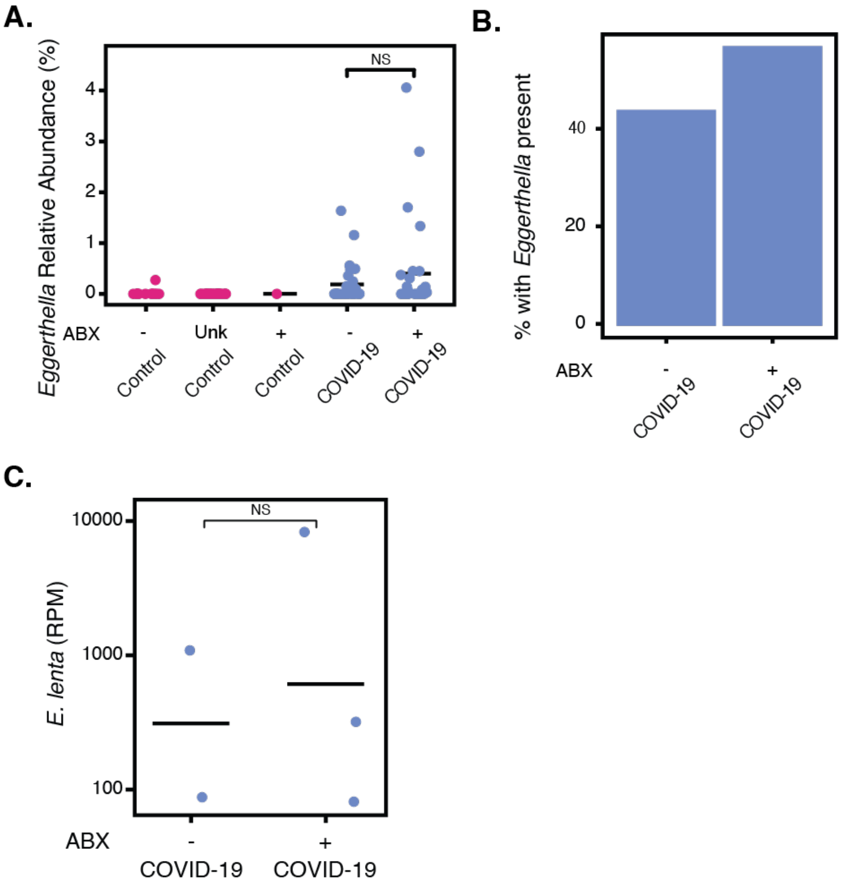


**Supplemental Fig. 5** The role of antibiotic exposure on *Eggerthella* species. **A** *Eggerthella* relative abundance in COVID-19 cases and controls based on antibiotic exposure status (ABX = antibiotic exposure, Unk = Unknown, not significant - NS, Kruskal-Wallis). **B** The percent of individuals with *Eggerthella* detected in their gut microbiome is shown for COVID-19 positive individuals.The reads per million (RPM) of **C** *Eggerthella lenta* (p = 1, Wilcoxon test) in positive COVID-19 individuals with and without antibiotic exposure.

**Suppl. Fig 6**

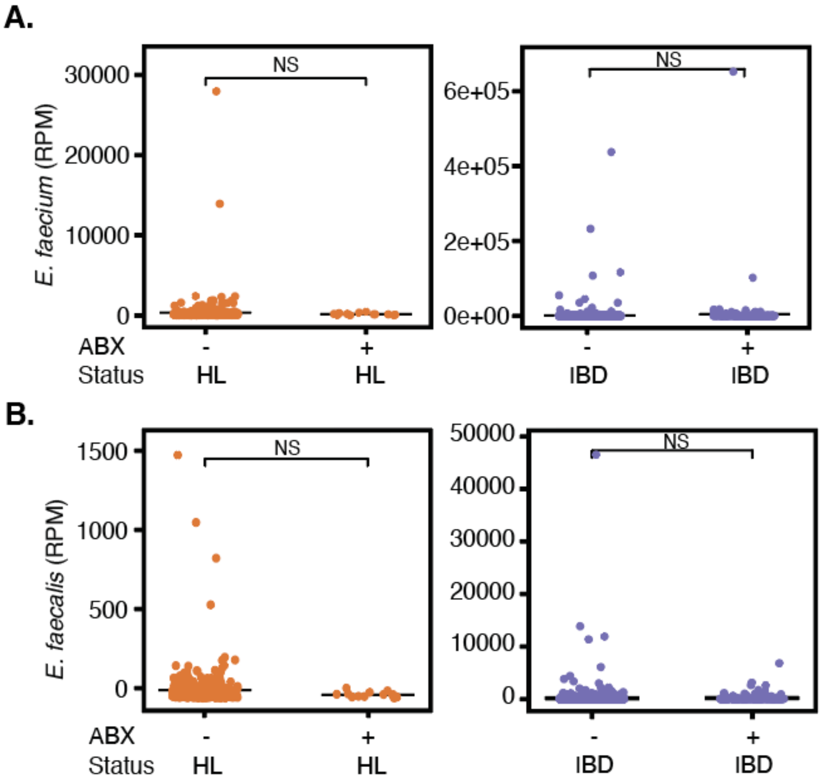


**Supplemental Fig. 6** The role of antibiotic treatment on the abundance of *Enterococcus* species in different disease states. A meta-analysis of USA studies with IBD patients and healthy controls was used to plot
**A** *Enterococcus faecium* (p = 1, Wilcoxon test) and **B** *Enterococcus faecalis* (p = 0.92, Wilcoxon test) (ABX = antibiotic treated, HL = healthy, IBD = irritable bowel disease, not significant - NS).
